# The Italian Hospital

**Published:** 1905-02-11

**Authors:** 


### EDITOR'S LETTER-BOX.

[Our Correspondents are reminded that prolixity is a great bar to publication, and that brevity of style and conciseness of statement greatly facilitate early insertion.]

### THE ITALIAN HOSPITAL.

"THE Secretary of the Italian Hospital presents his compliments to the Editor of THE HOSPITAL, and begs to forward copy of a resolution unanimously passed at the Annual General Meeting of Governors, held at the Hospital cn Saturday last, the 28th inst.

Resolved—That the cordial thanks of the Governors and Committee of Management be, and are hereby tendered to the Editor of THE HOSPITAL for his continued courtesy and greatly valued help to the Hospital during the past year."

January 31st, 1905.

